# Resveratrol inhibits Ca^2+^ signals and aggregation of platelets

**DOI:** 10.1186/s12199-020-00905-1

**Published:** 2020-11-07

**Authors:** Mikio Marumo, Kazumi Ekawa, Ichiro Wakabayashi

**Affiliations:** Department of Environmental and Preventive Medicine, Hyogo College of Medicine, Mukogawa-cho 1-1, Nishinomiya, Hyogo 663-8501 Japan

**Keywords:** Ca^2+^channels, Inositol trisphosphate, Store-operated Ca^2+^influx, Platelet aggregation, Resveratrol

## Abstract

**Background:**

Resveratrol has been shown to inhibit platelet aggregation. However, the mechanism for this action of resveratrol remains to be clarified. The purpose of this study was to elucidate the Ca^2+^-related mechanism for the inhibitory action of resveratrol on platelet aggregation.

**Methods:**

Ca^2+^ entry and subsequent aggregation of human platelets induced by different stimulants including thrombin, thapsigargin, and 1-oleoyl-2-acetylglycerol (OAG) were measured by the fluorescence method and light transmittance method, respectively. Each stimulant was added to a nominally Ca^2+^-free medium containing platelets, and then CaCl_2_ was added to the medium to induce Ca^2+^ influx into platelets.

**Results:**

Thapsigargin-induced Ca^2+^ entry into platelets and subsequent platelet aggregation were significantly inhibited in the presence of resveratrol at 6.25 μM or higher concentrations, while OAG-induced Ca^2+^ entry and subsequent platelet aggregation were not affected by resveratrol at concentrations up to 50 μM. In the nominally Ca^2+^-free medium, thrombin induced a small transient increase in intracellular Ca^2+^ concentrations, which was attenuated in the presence of resveratrol at 12.5 μM or higher concentrations. Thrombin-induced Ca^2+^ entry into platelets and subsequent platelet aggregation were significantly inhibited in the presence of resveratrol at 12.5 μM or higher concentrations.

**Conclusions:**

The results suggest that resveratrol inhibits thrombin-induced platelet aggregation through decreasing Ca^2+^ release from its stores and inhibiting store-operated Ca^2+^ influx into platelets.

## Introduction

The French paradox is based on epidemiological evidence that the incidence of ischemic heart disease in France is relatively low among western countries despite the saturated fat-rich diet of French people [1]. The French paradox is usually explained by the high consumption of wine, especially red wine, by French people [1]. Resveratrol, a non-flavonoid polyphenolic compound, is a stilbene derivative and is abundant in red wine [2]. Resveratrol can reduce the risk of cardiovascular disease through preventing the progression of atherosclerosis via its anti-oxidant actions. Resveratrol reduces the susceptibility of LDL cholesterol to oxidation, which initiates the formation of atherosclerotic plaque [3]. The viability of endothelium-derived nitric oxide, a crucial cardioprotective molecule, is increased by resveratrol [4]. In addition, platelet aggregation, which is a major process of arterial thrombus formation, is inhibited by resveratrol [5]. Health effects of resveratrol including improved antioxidant capacity and modulated neuroinflammation have been shown in human intervention trial studies [6].

A change in the intracellular Ca^2+^ concentration ([Ca^2+^]_i_) is a crucial signal for the regulation of cellular functions in various cells including platelets [7]. Platelet aggregation is induced by stimulants that cause elevation of [Ca^2+^]_i_. However, there have been few studies in which the effects of resveratrol on the Ca^2+^ signal and its associated functions of platelets were investigated. To the best of our knowledge, there has been only one research group that investigated the effects of resveratrol on Ca^2+^ signal in platelets: It was shown that resveratrol inhibited Ca^2+^ influx in platelets stimulated with thrombin and thapsigargin [8, 9], though corresponding platelet aggregation was not evaluated in those studies.

The purpose of this study was therefore to elucidate the effects of resveratrol in vitro on Ca^2+^ entry and subsequent aggregation of platelets. We used different stimulants of platelets, including thrombin, thapsigargin, and 1-oleoyl-2-acetylglycerol (OAG), that induce aggregation through different mechanisms for elevation of [Ca^2+^]_i_. Thrombin activates its receptors that are linked with the breakdown of phosphoinositides and induces Ca^2+^ influx mediated by two second messengers, inositol trisphosphate and diacylglycerol [10, 11]. Inositol trisphosphate induces the release of Ca^2+^ from its intracellular stores, resulting in store-operated Ca^2+^ influx (SOCI) through SOCI channels in the plasmalemma [12]. Thapsigargin induces Ca^2+^ influx by activation of SOCI channels [13], while OAG, an analog of diacylglycerol, induces Ca^2+^ influx through diacylglycerol-activated Ca^2+^ channels (non-SOCI channels), which are thought to be independent of intracellular Ca^2+^ stores [14, 15].

## Materials and methods

### Preparation of a washed-platelet suspension

Blood was obtained from healthy donors who had been medication-free for at least 10 days prior to the experiments. A platelet suspension was prepared from each donor in each experiment. This study was approved by the Ethics Committee of Hyogo College of Medicine (No. 1799), and the experimental procedures were in accordance with the Helsinki Declaration. Blood (18 ml) was rapidly transferred to a plastic tube containing 2 ml of 3.2% sodium citrate and mixed. The blood was then centrifuged at 150×*g* for 10 min, and the supernatant was obtained as platelet-rich plasma (PRP). PRP was subsequently mixed with 40 ml of Ca^2+^- and Mg^2+^-free Tyrode solution buffered by Hepes (NaCl 150 mM, KCl 5 mM, glucose 10 mM, HEPES 10 mM) (pH 7.4) and containing 1 mM EGTA, and the mixture was centrifuged at 150×*g* for 10 min. After the supernatant had been further centrifuged at 400×*g* for 5 min, the obtained pellet was suspended with 40 ml of the above Tyrode-Hepes solution and then further centrifuged at 400×*g* for 5 min. The pellet was suspended with 2 ml of Ca^2+^-free Tyrode solution (NaCl 150 mM, KCl 5 mM, MgCl_2_ 1 mM, glucose 10 mM, and HEPES 10 mM) (pH 7.4), and the resulting platelet suspension was used for the experiments within 2 h after blood collection. The concentration of platelets in its suspension used for each experiment was adjusted to be approximately 10^5^/μl. The numbers shown in the figures and tables are the numbers of experiments performed under experimental conditions using different stimulants.

### Measurement of [Ca^2+^]_i_

[Ca^2+^]_i_ was determined by using the fluorescent Ca^2+^ indicator fura-2. Washed platelets were loaded with fura-2/AM (final concentration, 5 μM) at 37 °C for 30 min. After loading, the platelets were washed once with Ca^2+^- and Mg^2+^-free Tyrode solution buffered by Hepes (NaCl 150 mM, KCl 5 mM, glucose 10 mM, HEPES 10 mM) (pH 7.4) and containing 1 mM EGTA, and they were resuspended in 2 ml of a Ca^2+^-free Tyrode solution buffered by Hepes (NaCl 150 mM, KCl 5 mM, MgCl_2_ 1 mM, glucose 10 mM, and HEPES 10 mM) (pH 7.4) (nominally Ca^2+^-free solution).

Fluorescence measurements were carried out with a dual-wavelength spectrofluorimeter (F-2500 Fluorescence Spectrophotometer, Hitachi High-Technologies Corporation, Tokyo, Japan) using a 0.4-ml cuvette maintained at 37 °C. The wavelengths used for excitation were 340 and 380 nm, and the wavelength used for emission was 510 nm. Fractional changes in [Ca^2+^]_i_ were determined by using a ratio (*R*) of fluorescence intensity (*F*) of F340/F380. The fluorescence after sequential addition of 0.25% Triton X-100 and EGTA (5 mM) to the platelet suspension provided the maximum fluorescence ratio (*R*_max_) and minimum fluorescence ratio (*R*_min_), respectively. [Ca^2+^]_i_ was calculated using the following formula [16]:

$$ {\left[{\mathrm{Ca}}^{2+}\right]}_{\mathrm{i}}=\left(\mathrm{R}\kern0.5em \hbox{-} \kern0.5em \mathrm{R}\min \right)/\left(\mathrm{R}\mathrm{max}\kern0.5em \hbox{-} \kern0.5em \mathrm{R}\right)\kern0.5em \mathrm{x}\kern0.5em \upbeta \kern0.5em \mathrm{x}\kern0.5em \mathrm{Kd}, $$

where *β* is the ratio of the emission fluorescence values at 380-nm excitation in the presence of Triton X-100 and EGTA, and *K*_d_, the dissociation constant for Ca^2+^, is 224. Ca^2+^ entry induced by thrombin, thapsigargin, or OAG was expressed as the net increase in [Ca^2+^]_i_ calculated by subtraction of the basal [Ca^2+^]_i_ level from the maximum [Ca^2+^]_i_ level after stimulation.

### Measurement of platelet aggregation

Platelet aggregation was measured using platelets suspended in Ca^2+^-free Tyrode solution buffered by Hepes (NaCl 150 mM, KCl 5 mM, MgCl_2_ 1 mM, glucose 10 mM, and HEPES 10 mM) (pH 7.4) and evaluated using an aggregometer (IMI PRP313M, IMI Co., Ltd., Saitama, Japan) that measures increases in light transmission through a cuvette (0.2 ml) containing a stirred platelet suspension. The light transmission through a washed-platelet suspension without any treatment and that through a suspended buffer not containing platelets were considered as 0 percent and 100 percent, respectively. The percentage of aggregation during the course of each experiment was calculated. The experimental conditions were the same as those for [Ca^2+^]_i_ measurement except for the volume of the cuvettes.

### Protocols for experiments to assess Ca^2+^ entry and platelet aggregation

Platelets were stabilized in nominally Ca^2+^-free medium in the cuvette for 3 min and then pretreated with various concentrations of resveratrol or a vehicle for 3 min. Platelets were then stimulated with each stimulant (thrombin [0.025 U/ml], thapsigargin [50 nM], or OAG [100 μM]). At 1 min after the addition of each stimulant, Ca^2+^ entry and aggregation were induced by adding CaCl_2_ (0.5 mM) to the cuvette.

### Drugs

Resveratrol (Sigma, St Louis, MO, USA) was dissolved in dimethylsulfoxide to make a stock solution of 50 mM and diluted with distilled water at the time of use. Thapsigargin (Sigma), OAG (Sigma), and fura-2/AM (Dojindo Laboratories, Kumamoto, Japan) were dissolved in dimethylsulfoxide to make stock solutions of 1 mM, 100 mM, and 5 mM, respectively, and stored at −80 °C. Bovine thrombin (Wako Pure Chemical) was dissolved in distilled water to make a stock solution of 1 U/μl and was stored at − 80 °C.

### Statistical analysis

Data are presented as means ± standard deviations. Statistical analysis was performed using analysis of variance followed by Scheffé *F* test. *P* values less than 0.05 were regarded as significant.

## Results

### Representative charts of measurement of platelet aggregation induced by different stimulants

**Fig. 1:**
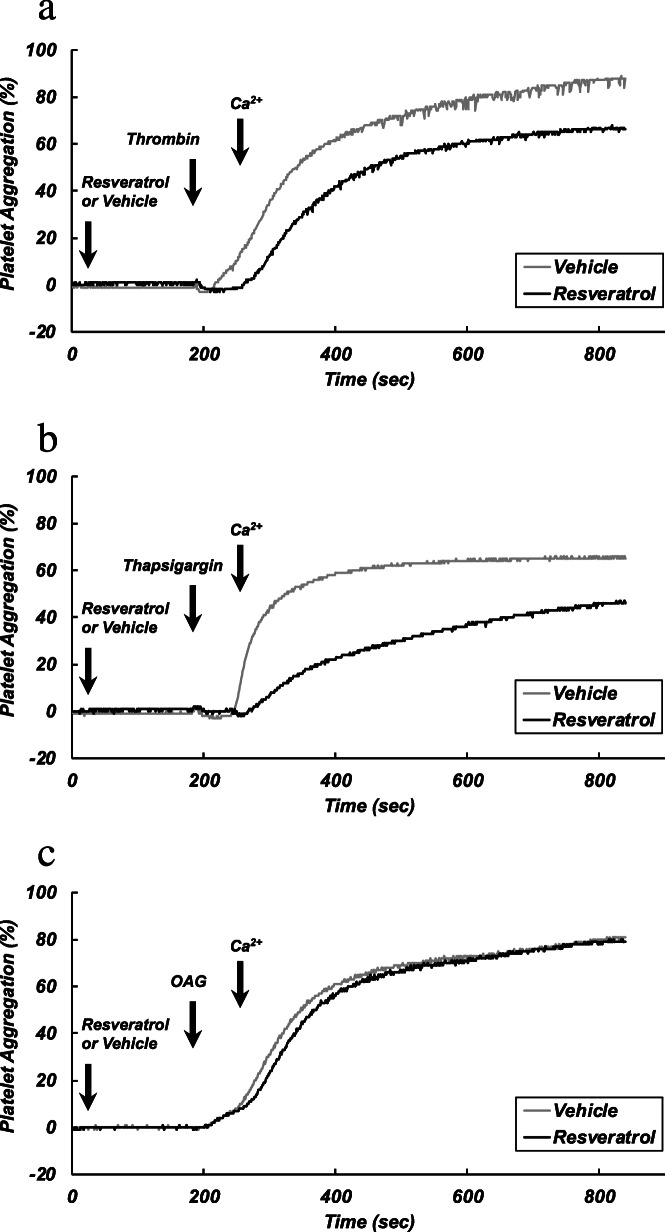
Representative charts of platelet aggregation. Washed platelets were incubated in nominally Ca^2+^-free solution. After stabilization, platelets were pretreated with resveratrol (12.5 μM) or a vehicle for 3 min. The platelets were then stimulated with thrombin (0.025 U/ml) (**a**), thapsigargin (0.1 μM) (**b**), or OAG (100 μM) (**c**). At 1 min after the addition of each stimulant, CaCl_2_ (0.5 mM) was added to the platelet suspension to induce platelet aggregation

Representative recordings of platelet aggregation by thrombin, thapsigargin, and OAG are shown in Fig. 1a, b, and c, respectively. In nominally Ca^2+^-free medium, OAG caused a slight aggregation, while no aggregation was induced by thrombin and thapsigargin. The addition of CaCl_2_ induced a large aggregation by each of the agonists. Pretreatment of platelets with resveratrol (12.5 μM) attenuated the aggregatory responses to CaCl_2_ in the presence of thrombin (Fig. 1a) and thapsigargin (Fig. 1b), while the response in the presence of OAG was not affected by resveratrol (Fig. 1c).

### Representative charts of measurements of intracellular Ca^2+^ concentrations in platelets stimulated with different agonists

**Fig. 2:**
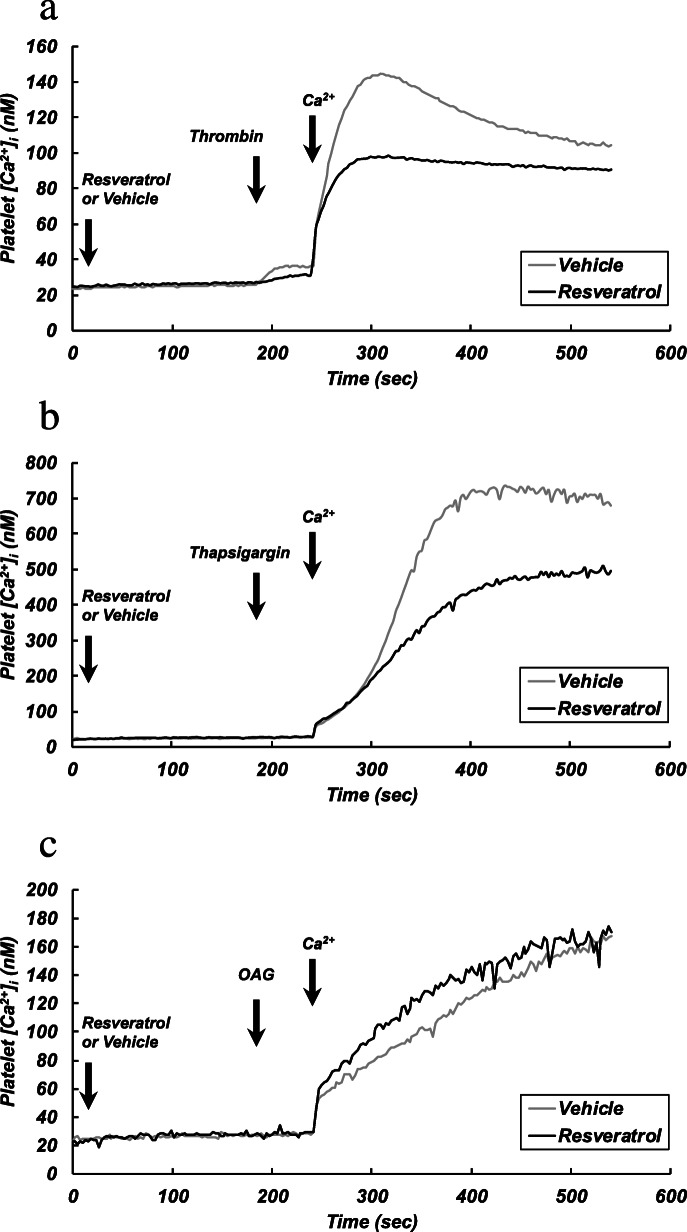
Representative charts of changes in [Ca^2+^]_i_. Washed platelets were incubated in nominally Ca^2+^-free solution. After stabilization, platelets were pretreated with resveratrol (12.5 μM) or a vehicle for 3 min. The platelets were then stimulated with thrombin (0.025 U/ml) (**a**), thapsigargin (0.1 μM) (**b**), or OAG (100 μM) (**c**). At 1 min after the addition of each stimulant, CaCl_2_ (0.5 mM) was added to the platelet suspension to induce Ca^2+^ entry

Representative recordings of [Ca^2+^]_i_ are shown in Fig. 2. In nominally Ca^2+^-free medium, thrombin induced a transient increase in [Ca^2+^]_i_, which was significantly smaller in the presence of resveratrol (12.5 μM) than in its absence [10.51 ± 3.94 nM (without resveratrol) vs. 5.54 ± 1.29 nM (with resveratrol), *p* < 0.05] (Fig. 2a). In nominally Ca^2+^-free medium, almost no changes in [Ca^2+^]_i_ were detected when platelets were stimulated with thapsigargin and OAG (Fig. 2b, c). In the presence of thrombin, thapsigargin or OAG, the addition of CaCl_2_ (final concentration of 0.5 mM) to the medium induced an increase in [Ca^2+^]_i_ (Ca^2+^ entry), which was much greater in thapsigargin-stimulated platelets (Fig. 2b) than in thrombin- or OAG-stimulated platelets (Fig. 2a, c). Pretreatment of platelets with resveratrol (12.5 μM) attenuated the increase in [Ca^2+^]_i_ in thrombin- or thapsigargin-stimulated platelets (Fig. 2a, b) but not in OAG-stimulated platelets (Fig. 2c).

### Effects of different concentrations of resveratrol on platelet aggregation

Effects of resveratrol at different concentrations on platelet aggregation induced by different stimulants are shown in Table 1. Thapsigargin-induced aggregation and thrombin-induced aggregation were significantly attenuated in the presence of resveratrol at concentrations from 6.25 to 50 μM in a concentration-dependent manner. Platelet aggregation induced by OAG was not significantly changed in the presence of resveratrol at concentrations up to 50 μM.

**Table 1:** Effects of different concentrations of resveratrol on platelet aggregation (upper lines) and Ca^2+^ entry into platelets (lower lines) induced by thrombin, thapsigargin, and OAG

Resveratrol (μM)	0	3.125	6.25	12.5	25	50
Thrombin						
Aggregation (%)	81.83 (4.02)	73.38 (3.46)	66.25 (5.50)*	58.33 (12.09)**	40.8 (6.53)**	21.75 (9.57)**
Ca^2+^ entry (nM)	125.34 (29.13)	104.73 (19.32)	101.63 (25.94)	92.59 (18.37)*	83.65 (4.98)**	63.52 (11.55)**
Thapsigargin						
Aggregation (%)	69.88 (3.08)	71.00 (5.18)	63.86 (7.40)**	49.17 (10.61)**	38.00 (9.17)**	20.50 (8.34)**
Ca^2+^ entry (nM)	604.78 (128.07)	465.95 (126.16)	396.62 (129.83)**	395.69 (70.90)**	351.37 (37.91)**	320.73 (68.26)**
OAG						
Aggregation (%)	76.60 (4.40)	75.00 (3.00)	77.33 (2.31)	74.00 (4.42)	70.50 (3.41)	67.00 (6.90)
Ca^2+^ entry (nM)	93.31 (16.64)	90.57 (6.92)	95.54 (5.45)	88.64 (12.48)	79.67 (11.28)	77.19 (12.11)

Data are means (%) with standard deviations indicated in parentheses. Platelets were stimulated with thrombin (0.025 U/ml), thapsigargin (0.1 μM), and OAG (100 μM). Asterisks denote significant differences (*p < 0.05; **p < 0.01) from the control in the absence of resveratrol. *N* = 5-9

### Effects of different concentrations of resveratrol on Ca^2+^ entry into platelets

Effects of resveratrol at different concentrations on Ca^2+^ entry induced by different stimulants are also shown in Table 1. Thapsigargin-induced Ca^2+^ entry and thrombin-induced Ca^2+^ entry were significantly inhibited by resveratrol in concentration-dependent manners at concentrations from 6.25 to 50 μM and concentrations from 12.5 to 50 μM, respectively. Ca^2+^ entry induced by OAG was not significantly affected by resveratrol at concentrations up to 50 μM.

### Effects of different concentrations of resveratrol on thrombin-induced transient increase in [Ca^2+^]_i_ in nominally Ca^2+^-free medium

Since the thrombin (0.025 U/ml)-induced transient increase in [Ca^2+^]_i_ in nominally Ca^2+^-free medium was small (Fig. 2a), the effect of resveratrol on the transient increase in [Ca^2+^]_i_ induced by a higher concentration (0.1 U/ml) of thrombin was examined. The thrombin-induced transient increase in [Ca^2+^]_i_ in nominally Ca^2+^-free medium was significantly inhibited by resveratrol in a concentration-dependent manner at concentrations from 12.5 to 50 μM (means ± standard deviations [nM]: 24.5 ± 2.9 [control]; 22.0 ± 1.8 [resveratrol 6.25 μM]; 20.1 ± 1.5 [resveratrol 12.5 μM, *p* < 0.05]; 19.0 ± 1.0 [resveratrol 25 μM, *p* < 0.01]; 17.1 ± 1.9 [resveratrol 50 μM, *p* < 0.01]).

## Discussion

In this study, the effects of resveratrol in vitro on aggregation of platelets and its related Ca^2+^ signal were investigated. We showed for the first time that resveratrol inhibits thrombin-induced release of Ca^2+^ from its stores and store-operated Ca^2+^ entry but not Ca^2+^ entry induced by OAG, an analog of diacylglycerol. Inhibitory effects of resveratrol on aggregation of platelets corresponding to the Ca^2+^ entry were also demonstrated in the present study. Since alcohol (ethanol) is known to also inhibit Ca^2+^ entry and subsequent aggregation of platelets [17–19], red wine that contains resveratrol as well as ethanol may have a potent inhibitory action on platelet aggregation. This may explain the cardioprotective effect of red wine through its antithrombotic actions [20].

**Fig. 3:**
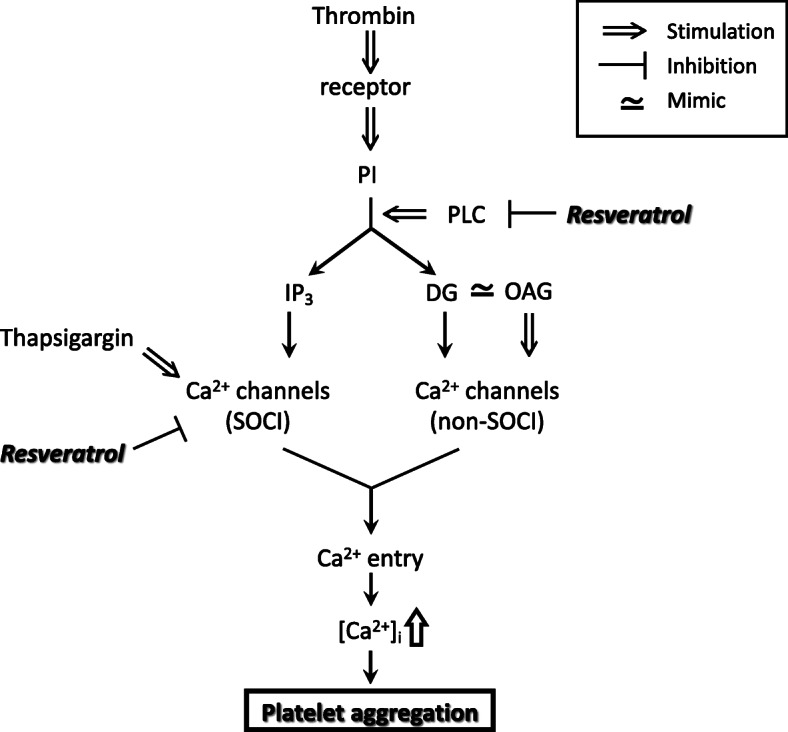
A scheme of the hypothesized mechanism for inhibition of platelet aggregation by resveratrol suggested by the results of this study. DG, diacylglycerol; IP_3_, inositol trisphosphate; OAG, 1-oleoyl-2-acetylglycerol; PI, phosphoinositides; PLC, phospholipase C; SOCI, store-operated Ca^2+^ influx

Thrombin activates phospholipase C, resulting in hydrolysis of phosphoinositides and production of two intracellular messengers, inositol trisphosphate and diacylglycerol. Inositol trisphosphate causes release of Ca^2+^ from its stores and subsequent Ca^2+^ depletion in the stores, resulting in activation of SOCI channels and induction of Ca^2+^ influx through the plasmalemma [12]. Our finding of an inhibitory action of resveratrol on thrombin-induced increase in [Ca^2+^]_i_ in a nominally Ca^2+^-free solution, which corresponds to inositol trisphosphate-induced release of Ca^2+^ from its stores, agrees with the finding in a previous study that phospholipase C activity in platelets was inhibited by resveratrol [21]. Thapsigargin is a stimulant of SOCI channels and induces increase in [Ca^2+^]_i_ independently of inositol trisphosphate. Therefore, the findings of inhibitory effects of resveratrol on increase in [Ca^2+^]_i_ and aggregation of platelets induced by thapsigargin suggest that resveratrol inhibits SOCI channels. Thus, resveratrol is thought to hamper two sites contributing to Ca^2+^-related signal transduction, phospholipase C and SOCI channels of platelets, resulting in a decrease in thrombin-induced elevation of [Ca^2+^]_i_ and attenuation of subsequent aggregation. Diacylglycerol, the other product of hydrolysis of phosphoinositides, also induces transmembraneous Ca^2+^ influx through Ca^2+^ channels that are independent of Ca^2+^ stores (non-SOCI channels) [22]. However, an increase in [Ca^2+^]_i_ and aggregation induced by OAG, a mimic of diacylglycerol, were not affected by resveratrol. When platelets are stimulated by thrombin, two different channels are activated through inositol trisphosphate and diacylglycerol. Thus, resveratrol is thought to selectively inhibit Ca^2+^ entry, namely, to inhibit only SOCI, in platelets. Figure 3 summarizes the hypothesized mechanism for the effect of resveratrol on thrombin-induced platelet aggregation suggested by the results of the present study: When platelets are stimulated with thrombin, resveratrol inhibits phospholipase C and SOCI channels separately, resulting in less elevation of [Ca^2+^]_i_ and thereby attenuation of the Ca^2+^-dependent aggregatory response of platelets.

Resveratrol has been demonstrated to show inhibitory action on platelet aggregation both in vitro and in vivo [23]. However, the concentrations of resveratrol that were reported to inhibit platelet function in vitro differed in previous studies, and the range of the reported effective concentrations was large (0.05 ~ 100 μM) [9, 21, 24–26]. Moreover, the sensitivity of platelet aggregation to resveratrol was reportedly lower in whole blood than in washed platelets. Therefore, other factors in blood including erythrocytes and leukocytes are suspected to interfere with the action of resveratrol on platelet aggregation [27].

Further studies including experiments to test the effects of administration of resveratrol on platelet aggregability in vivo are needed to clarify the reason for the discrepancy regarding the effective concentrations of resveratrol and to determine whether the inhibitory action of resveratrol on platelet aggregation is clinically significant. Moreover, the detailed mechanism for inhibition of Ca^2+^-related signals in platelets remains to be clarified. Since resveratrol has a potent antioxidant action, it would be interesting to elucidate the relation of antioxidant action of resveratrol to Ca^2+^ signals in platelets. In fact, resveratrol has been shown to stimulate nitric oxide production in platelets [28] and to increase the viability of nitric oxide in vascular endothelial cells [4]. In addition, Ca^2+^ entry through SOCI channels, which are also called capacitative Ca^2+^ channels, was inhibited by nitric oxide in vascular smooth muscle cells [29] and endothelial cells [30]. Therefore, it is speculated that resveratrol inhibits SOCI via an increase in nitric oxide in platelets, and this speculation should be examined in future studies.

## Conclusion

Resveratrol inhibits thrombin-induced platelet aggregation through decreasing Ca^2+^ release from its stores and inhibiting SOCI into platelets. Diacylglycerol-induced Ca^2+^ entry through non-SOCI channels is not affected by resveratrol.

## Data Availability

No additional data are available.

## Abbreviations

[Ca^2+^]_i_: Intracellular Ca^2+^ concentrations
DG: Diacylglycerol
IP_3_: Inositol trisphosphate
OAG: 1-Oleoyl-2-acetylglycerol
PI: Phosphoinositides
SOCI: Store-operated Ca^2+^ influx
